# Congenital monocular elevation deficiency associated with a novel *TUBB3* gene variant

**DOI:** 10.1136/bjophthalmol-2019-314293

**Published:** 2019-07-13

**Authors:** Mervyn G Thomas, Gail D E Maconachie, Cris S Constantinescu, Wai-Man Chan, Brenda Barry, Michael Hisaund, Viral Sheth, Helen J Kuht, Rob A Dineen, Sreemathi Harieaswar, Elizabeth C Engle, Irene Gottlob

**Affiliations:** 1 Ulverscroft Eye Unit, Department of Neuroscience, Psychology and Behaviour, University of Leicester, Leicester, UK; 2 Department of Neurology, University of Nottingham, Nottingham, UK; 3 Howard Hughes Medical Institute, Chevy Chase, Mayland, United States; 4 Department of Neurology, Boston Children's Hospital, Boston, Massachusetts, United States; 5 Department of Radiology, University of Nottingham, Nottingham, UK; 6 Department of Radiology, University Hospitals of Leicester NHS Trust, Leicester, UK; 7 Departments of Neurology and Ophthalmology, Boston Children’s Hospital, Boston, Massachusetts, United States; 8 Departments of Neurology and Ophthalmology, Harvard Medical Schoool, Boston, Massachusetts, United States

**Keywords:** monocular elevation deficiency, double elevator palsy, TUBB3, congenital fibrosis of extraocular muscles, CFEOM

## Abstract

**Background:**

The genetic basis of monocular elevation deficiency (MED) is unclear. It has previously been considered to arise due to a supranuclear abnormality.

**Methods:**

Two brothers with MED were referred to Leicester Royal Infirmary, UK from the local opticians. Their father had bilateral ptosis and was unable to elevate both eyes, consistent with the diagnosis of congenital fibrosis of extraocular muscles (CFEOM). Candidate sequencing was performed in all family members.

**Results:**

Both affected siblings (aged 7 and 12 years) were unable to elevate the right eye. Their father had bilateral ptosis, left esotropia and bilateral limitation of elevation. Chin up head posture was present in the older sibling and the father. Bell’s phenomenon and vertical rotational vestibulo-ocular reflex were absent in the right eye for both children. Mild bilateral facial nerve palsy was present in the older sibling and the father. Both siblings had slight difficulty with tandem gait. MRI revealed hypoplastic oculomotor nerve. Left anterior insular focal cortical dysplasia was seen in the older sibling. Sequencing of *TUBB3* revealed a novel heterozygous variant (c.1263G>C, p.E421D) segregating with the phenotype. This residue is in the C-terminal H12 α-helix of β-tubulin and is one of three putative kinesin binding sites.

**Conclusion:**

We show that familial MED can arise from a *TUBB3* variant and could be considered a limited form of CFEOM. Neurological features such as mild facial palsy and cortical malformations can be present in patients with MED. Thus, in individuals with congenital MED, consideration may be made for *TUBB3* mutation screening.

## Introduction

Monocular elevation deficiency (MED), previously called double elevator palsy, is a rare disorder characterised by inability to elevate one eye above the horizontal plane.^1^ The term ‘double elevator palsy’ was used since it was considered to be a congenital palsy of the two ipsilateral elevators (inferior oblique and superior rectus (SR)).^1^ MED is thought to arise due to three potential mechanisms: SR paresis, inferior rectus (IR) restriction or a unilateral supranuclear abnormality.^2^ MRI evidence suggests the latter is the most likely pathophysiological mechanism based on normal extraocular muscles and oculomotor nerves in most patients with MED.^3^

A genetic cause has been hypothesised in identical twins with MED on the same side and a preserved Bell’s phenomenon, thus implicating a supranuclear defect.^4^ Due to the overlap of MED with congenital cranial dysinnervation disorders (CCDDs), Volk *et al* screened four patients with MED for *CHN1* mutations; however, no mutations were detected.^5^ Doherty *et al* described the CFEOM3 phenotype which is a non-progressive autosomal dominant eye movement disorder characterised by variable expression of ptosis and restrictive external ophthalmoplegia; some family members had only absence of upgaze.^6^ CFEOM3 was subsequently reported to result from recurrent mutations in *TUBB3*.^7^ We identified a family with two siblings that presented with MED and their father who presented with CFEOM, and performed candidate gene screening of autosomal dominant CFEOM genes to identify the causative genetic variant for the phenotype.

## Methods

**Figure 1:**
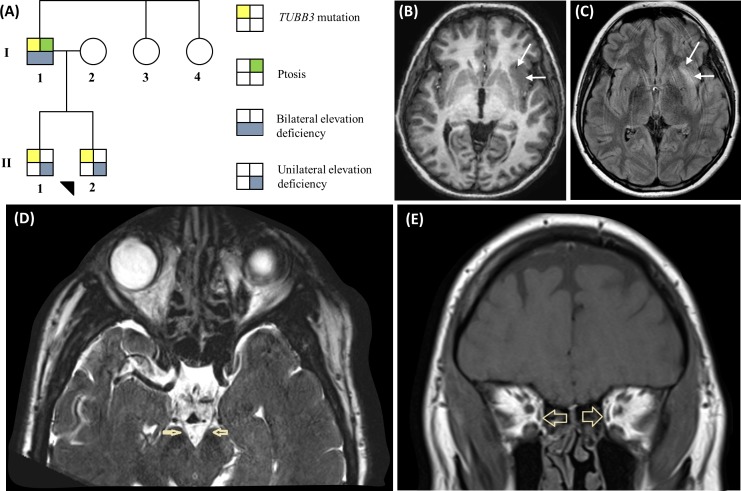
(A) Pedigree of family with *TUBB3* mutation. (B and C) MRI from F1:II-1 showing the left anterior insular cortical malformation. (B) Axial T1-weighted image showing thickening of the anterior insular cortex (arrows) with indistinct grey–white matter interface, and (C) corresponding T2 hyperintensity (arrows) on the T2-weighted fluid-attenuated inversion recovery image. (D) Axial T2-weighted image showing thread-like oculomotor nerves (arrows) in F1:I-1. (E) Coronal T1-weighted image showing small medial recti (arrows) in F1:I-1

The pedigree of the family with MED is shown in figure 1A. The clinical characteristics of the family are shown in table 1.

**Table 1:** Clinical characteristics of family with *TUBB3* mutation

						Corrected VA					Limited horizontal duction				Limited vertical duction			
ID											R Eye		L Eye		R Eye		L Eye	
	Gender	Age	Ref (RE)	Ref (LE)	AHP (D)	RE	LE	Type of strabismus	Binocularity	Ptosis	Ab	Ad	Ab	Ad	Up	Dn	Up	Dn
F1:II-2	M	7	+3.50	+3.50	Right Tilt	0.28 (6/12+1)	0.28 (6/12+1)	Right HoT	150”	Nil	0	0	0	0	−3	0	0	0
F1:II-1	M	12	+4.50	+5.00	Chin up	0.30 (6/12)	0.22 (6/9–1)	Right HoT	150”	Nil	0	0	0	0	−3	0	0	0
F1:I-1	M	39	−9.38	−13.00	Chin up	0.50 (6/18)	1.00 (6/60)	Left ET	Nil	Bilateral	0	0	−2	0	−2	−2	−2	−2

AHP (D), anomalous head posture (for distance); Ab, abduction; Ad, adduction; ET, esotropia; HoT, hypotropia; LE, left eye; RE, right eye; Ref, refraction (spherical equivalent); VA, visual acuity in logMAR (and Snellen).

All family members underwent a detailed ophthalmological and neurological examination. We assessed for Bell’s phenomenon and rotational vestibulo-ocular reflex to assess if there was a supranuclear defect. MRI scans were performed using a 3-Tesla Philips Achieva scanner in all three affected subjects. Eye movement recordings were obtained, in individual F1:II-1, using an infrared pupil tracker (EyeLink II; SR Research, Ottawa, Canada: sample rate=500 Hz, spatial resolution=0.01° RMS) as previously described.^8 9^ A series of saccadic tasks were used to obtain the saccadic main sequence and plot the relationship between peak saccadic velocity and saccadic amplitude.

Saliva samples (Oragene DNA sample Collection Kit (OG-500; DNA Genotek, Ottawa, Ontario, Canada)) were obtained from all family members. DNA was extracted from the saliva samples. All coding exons and intron–exon boundaries of *TUBB3* and *TUBB2B* and exons 8, 20 and 21 of *KIF21A* were sequenced as previously reported.^7 10 11^ Primer sequences are available on request.

## Results

**Figure 2:**
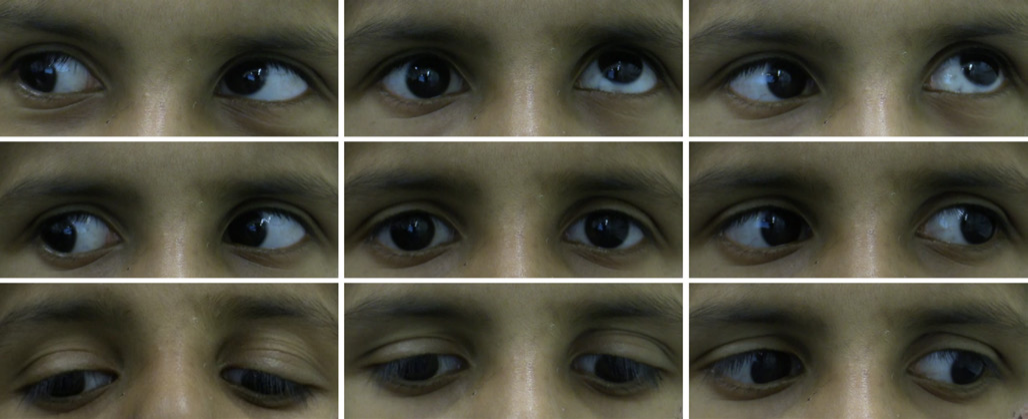
Nine positions of gaze showing monocular elevation deficiency of the right eye in F1:II-1.

**Figure 3:**
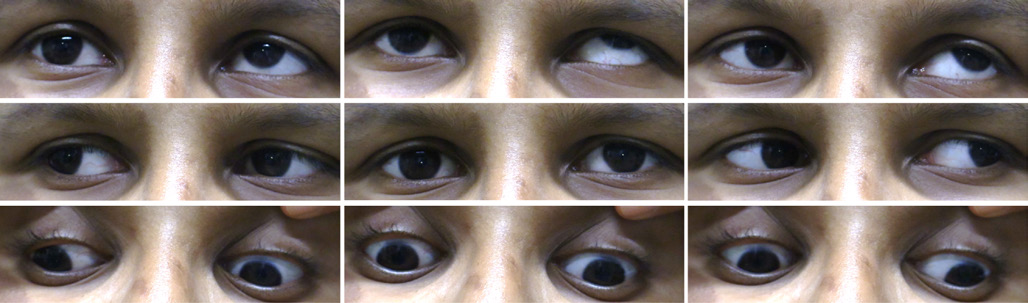
Nine positions of gaze showing monocular elevation deficiency of the right eye in F1:II-2.

**Figure 4:**
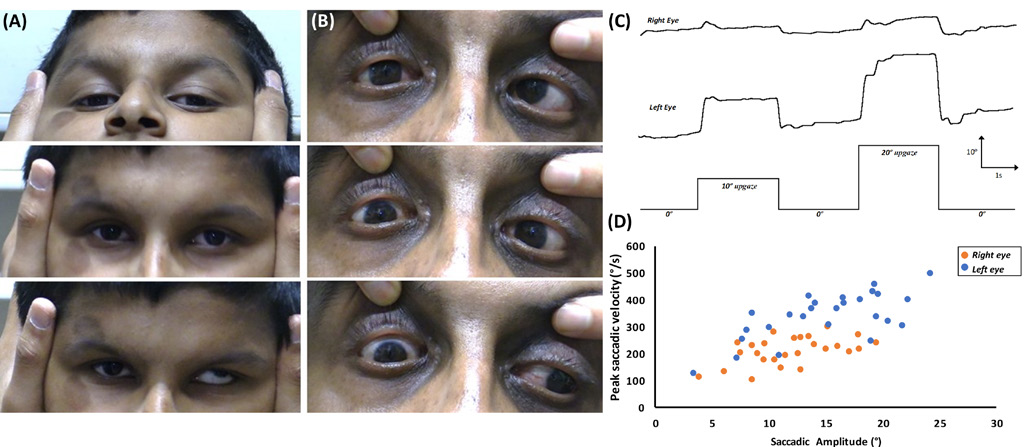
(A) Rotational vestibulo-ocular reflex during pitch rotations in F1:II-1 shows lack of upward movement of the right eye. (B) Abduction of the left eye is noted on downgaze which could be a pathological synkinetic movement or due to a tight inferior rectus in F1:II-1 (upper panel=upgaze; middle panel=primary position; lower panel=downgaze). (C) Eye movement recordings in F1:II-1 showing poor elevation of the right eye from primary position. Deflection upwards represents movement of the eyes upwards, while deflection downwards represents movement of the eyes downwards. X-axis=time (in seconds); Y-axis=eye rotation (in degrees). (D) Plot of the peak saccadic velocity in relation to the saccadic amplitude, showing reduced saccadic velocity of the right eye compared with the left for upgaze.

The proband (F1:II-2), aged 7 years, was referred by the optician to the paediatric ophthalmology department (Leicester Royal Infirmary, UK) due to inability to elevate the right eye, reduced visual acuity (VA) and poor attention span. He was accompanied by his father (F1:I-1) and older brother (F1:II-1). There was no significant medical history. On examination, F1:II-2 was unable to elevate the right eye above the horizontal plane, consistent with a diagnosis of MED (figure 2). VA was reduced to 0.28logMAR (Snellen equivalent 6/12+1) in both eyes. Stereopsis was 150 s of arc and a head tilt to the right was observed. Incidentally, his older brother, aged 12 years, was noted to have MED of the right eye (figure 3), reduced VA and chin up head posture (table 1). Both children demonstrated absent right Bell’s phenomenon. Vertical rotational vestibulo-ocular reflex (r-VOR) did not produce upward movement past the midline (figure 4A). A prominent right lower lid skin crease, in both siblings, was seen on attempted up gaze (figures 2 and 3). F1:I-1 had bilateral ptosis, left esotropia, bilateral limitation of elevation and a chin up head posture, consistent with the diagnosis of CFEOM (figure 4B).

The affected members had been diagnosed as having mild learning difficulties, and had been or were in special education. On neurological examination, mild ataxia demonstrated by slight difficulty with tandem walking. F1:II-1 and F1:I-1 had mild bilateral facial nerve palsy with slightly flat nasolabial folds and a horizontal smile. F1:I-1 demonstrated decreased sensation to all modalities with depressed deep tendon reflexes (upper and lower extremities); however, nerve conduction studies were normal. MRI scans were successful in F1:I-1 and F1:II-1; however, the scan protocol was incomplete in F1:II-2 due to movement artefacts. Corpus callosum, olfactory sulci/bulbs and basal ganglia were normal. Hypoplastic oculomotor nerves were seen in both F1:I-1 (figure 1D) and F1:II-1. The anterior commissure was small in F1:II-1. He was also noted to have focal cortical dysplasia involving the left anterior insular cortex (figure 1B, C), which remained unchanged after 1 year. Tortuous basilar artery and intracranial internal carotid artery were seen in F1:II-2 and F1:I-1, respectively. Extraocular muscle interpretation was limited in both children due to movement artefacts. F1:I-1 had bilateral small medial (figure 1E) and superior recti.

Eye movement recordings showed limitation of elevation from primary position (figure 4C). Plots of peak saccadic velocity against saccadic amplitude showed a reduced saccadic velocity on upgaze of the right eye in F1:II-1 compared with his left eye saccadic velocity (figure 4D).

**Figure 5:**
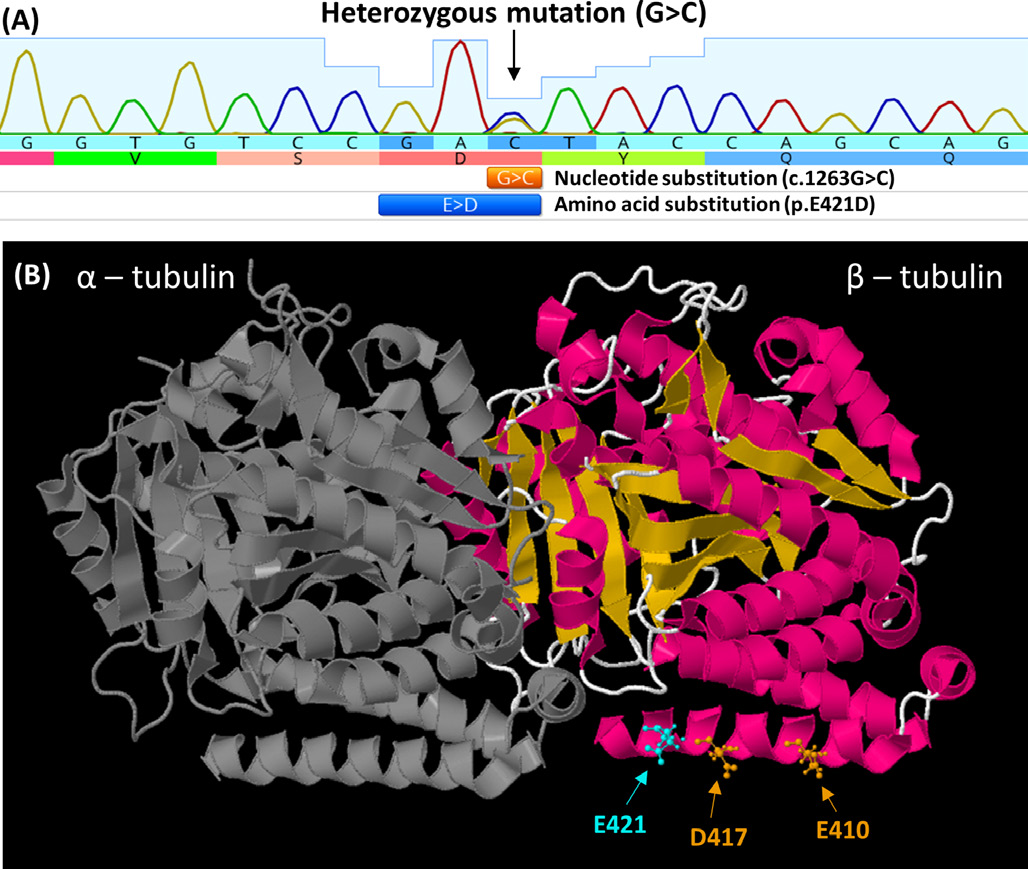
(A) Electropherogram from F1:II-2, showing G>C nucleotide substitution in *TUBB3*. This results in the amino acid substituition glutamic acid (E) to aspartic acid (D) at amino acid position 421. (B) The location of the E421 residue (cyan) mapped on the solved protein structure of *TUBB3* (PDB ID: 5IJ0). This is adjacent to previously reported mutations at residues 417 and 410 associated with congenital fibrosis of extraocular muscles (orange). All three mutations are located on the H12 α-helix and predicted to be direct binding sites required for kinesin binding to the microtubule polymer.

Sanger sequence analysis identified a novel *TUBB3* variant (c.1263G>C, p.E421D) that segregated appropriately in the family (figure 5A). The variant is absent from public databases (gnomAD (genome Aggregation Database), dbSNP151), alters an amino acid residue within a functional protein domain (Tubulin/FtsZ, C-terminal, β-tubulin) and is predicted to be damaging (based on in silico analyses: PolyPhen-2, MutationTaster2 and CADD models).

## Discussion

In this study, we show that MED in this family is a CCDD and a subset of CFEOM that can arise from *TUBB3* variants. The CFEOM3 phenotype can have variable expression with bilateral or unilateral oculomotility defects ranging from complete ophthalmoplegia to mild restrictions, including isolated bilateral upgaze palsy.^6 7^ *TUBB3* mutations have also been described in patients with Moebius syndrome.^12^ The E421D syndrome described here has variable expression which can include bilateral or unilateral ophthalmoplegia, ptosis, pathological synkinesis, mild learning difficulty, facial nerve palsy, focal cortical dysplasia and mild ataxia.

*TUBB3* mutations can cause isolated or syndromic CFEOM3 (OMIM 600638) or malformations of cortical development (OMIM 614039).^7 13^ Two mutations (G71R and G98S) have been described to be associated with both malformations of cortical development and syndromic CFEOM.^13^ The variant presented in this study (E421D) represents the third variant where both phenotypes are seen. To date, there are just over 20 *TUBB3* mutations reported in the literature. The c.1263G>C variant is predicted to cause a p.E421D amino acid substitution in the C-terminal H12 α-helix of β-tubulin (figure 5B). The E421 residue is conserved across β-tubulin isotypes from yeast to humans. Based on an in vitro study, E410, D417 and E421 were reported to be the three β-tubulin residues with which kinesin motors directly interact.^7 14^ While E410 and D417 were previously reported to be altered by CFEOM mutations,^7^ this is the first *TUBB3* variant altering E421. Notably, a variant altering this residue (E421K) was described and characterised in *TUBB2B*.^11^ Interestingly, this variant in *TUBB2B* is associated with CFEOM and polymicrogyria, while most *TUBB2B* mutations are associated with polymicrogyria without CFEOM (OMIM 610031). Based on data from the D417H mutation,^7 14^ we hypothesise that the E421D amino acid substitution also reduces interactions with microtubule-associated proteins thus affecting cytoskeletal architecture and motor transport and resulting in the phenotype observed.

Three mechanisms have been proposed to cause MED: IR restriction, SR paresis or a supranuclear defect.^2 3^ To isolate the neuroanatomical origins of MED, we assessed Bell’s phenomenon and vertical r-VOR. Bell’s phenomenon and r-VOR were absent in the right eye for both siblings, which suggests that a supranuclear defect is unlikely. A prominent right lower lid skin crease, more pronounced on upgaze, was seen in both siblings which can be a sign of IR contracture.^15 16^ The reduction in saccadic velocity in the right eye compared with left for upgaze could be a sign of SR paresis.

## Conclusion

In this report, we show that familial MED can arise due to *TUBB3* gene variants. This form of MED can be considered a limited form of CFEOM that arises due to a lower motor neuron developmental abnormality. Therefore, *TUBB3* genetic testing may be considered in patients presenting with familial MED or MED (non-familial MED associated with other mild deficits in ocular rotation). The TUBB3 E421 residue is the third kinesin interaction site to be altered in CFEOM, and the resulting E421D syndrome can include CFEOM, mild learning difficulty, facial nerve palsy, focal cortical dysplasia and mild ataxia.
